# How well do final year undergraduate medical students master practical clinical skills?

**DOI:** 10.3205/zma001057

**Published:** 2016-08-15

**Authors:** Sylvère Störmann, Melanie Stankiewicz, Patricia Raes, Christina Berchtold, Yvonne Kosanke, Gabrielle Illes, Peter Loose, Matthias W. Angstwurm

**Affiliations:** 1 Klinikum der Universität München, Medizinische Klinik und Poliklinik IV, München, Germany

**Keywords:** practical examination, OSCE, physical examination, clinical skills

## Abstract

**Introduction: **The clinical examination and other practical clinical skills are fundamental to guide diagnosis and therapy. The teaching of such practical skills has gained significance through legislative changes and adjustments of the curricula of medical schools in Germany. We sought to find out how well final year undergraduate medical students master practical clinical skills.

**Methods:** We conducted a formative 4-station objective structured clinical examination (OSCE) focused on practical clinical skills during the final year of undergraduate medical education. Participation was voluntary. Besides the examination of heart, lungs, abdomen, vascular system, lymphatic system as well as the neurological, endocrinological or orthopaedic examination we assessed other basic clinical skills (e.g. interpretation of an ECG, reading a chest X-ray). Participants filled-out a questionnaire prior to the exam, inter alia to give an estimate of their performance.

**Results: **214 final year students participated in our study and achieved a mean score of 72.8% of the total score obtainable. 9.3% of participants (n=20) scored insufficiently (<60%). We found no influence of sex, prior training in healthcare or place of study on performance. Only one third of the students correctly estimated their performance (35.3%), whereas 30.0% and 18.8% over-estimated their performance by 10% and 20% respectively.

**Discussion: **Final year undergraduate medical students demonstrate considerable deficits performing practical clinical skills in the context of a formative assessment. Half of the students over-estimate their own performance. We recommend an institutionalised and frequent assessment of practical clinical skills during undergraduate medical education, especially in the final year.

## Zusammenfassung

**Einleitung: **Die körperliche Untersuchung und klinisch-praktische Fertigkeiten sind wesentliche ärztliche Fähigkeiten, mit deren Hilfe die Diagnostik und Therapie von Krankheiten gesteuert werden. Durch curriculare Veränderungen wird der praktischen Ausbildung ein hoher Stellenwert eingeräumt. Wie gut beherrschen also Studierende im Praktischen Jahr (PJ) klinisch-praktische Fertigkeiten?

**Methoden: **Wir führten eine freiwillige mündlich-praktische Prüfung mittels OSCE bei Studierenden im PJ durch. Inhalte der Prüfung waren u.a. die körperliche Untersuchung (Herz, Lunge, Abdomens, Gefäßsystem, Lymphsystem; neurologische, endokrinologische bzw. orthopädische Untersuchung) sowie elementare praktische Fertigkeiten (etwa EKG-Interpretation, Basis-Befundung Röntgen-Thorax). Die Teilnehmer füllten zudem vor Beginn der Prüfung einen Fragebogen aus, u.a. zur Einschätzung der eigenen Leistung.

**Ergebnisse:** Insgesamt 214 PJ-Studierende nahmen teil und erreichten 72,8% der erreichbaren Punktzahl. Eine nicht ausreichende Leistung (<60%) zeigten 9,3% der Teilnehmer (n=20). Geschlecht, vorangegangene Ausbildung in einem Gesundheitsberuf sowie Studienort hatten keinen Einfluss auf die Leistung. Im Mittel schätzten sich die Studierenden 0,5 Notenstufen besser. 35,3% der Teilnehmer vermochten ihre Leistung richtig einzuschätzen. 30,0% überschätzten ihr Ergebnis um eine Notenstufe, 18,8% um zwei oder mehr Notenstufen.

**Diskussion: **Studierende im Praktischen Jahr zeigen deutliche Defizite bei der Durchführung klinisch-praktischer Fertigkeiten im Rahmen einer mündlich-praktischen Prüfung. Dabei überschätzt knapp die Hälfte der Studierenden die eigene Leistung. Eine institutionalisierte, regelhafte Prüfung der mündlich-praktischen Fähigkeiten im Praktischen Jahr erscheint daher notwendig.

## 1. Introduction

The basic clinical examination is a fundamental skill of physicians that facilitates diagnosis and therapy [1], [2], [3]. Technical progress has changed the practice of medicine, which increasingly relies on laboratory assessments, diagnostic imaging, and other sources of technical examination. Some authors mourn that this has led to a deterioration of the ability to perform a systematic and focused hands-on clinical examination [4], [5]. Medical students and young physicians alike demonstrate deficits in performing a clinical examination [6], [7]. A legislative reform in Germany in 2002 (Medical Licensure Act) pushed the faculties to develop new curricula with an emphasis on practical training [8], [9], [10], [11], [12], [13], [14]. By specifically defining the type and amount, this law formalised clinical practical teaching and its curricular design [15]. This lead to novel teaching concepts as well as whole curricula centred on medical skills [16], [17], [18], [19], [20]. An intensive training of practical clinical skills during the first years of undergraduate medical education aims to prepare students well for their future role as physicians; the final year is an important landmark in undergraduate training and consists mainly of practical exercise of previously and newly acquired skills [21]. The Medical Curriculum (MeCuM) integrates clinical training into pre-clinical courses during year 2 of undergraduate studies, starting with history taking. Through a variety of diverse formats (lectures, bedside teaching, peer teaching, and blended learning) students learn the theoretical basis of the clinical examination and have the opportunity to put it into practice. Longitudinal internships in general medical practices and frequent bedside courses allow practical exercise and feedback discussions steer the learning. As students progress, further practical clinical skills are taught in the context of their respective system (such as writing and reading an ECG as part of the cardiovascular teaching block, interpretation of a chest X-ray during the respiratory teaching block). Higher-level skills such as clinical decision-making are part of the formation during the final year when students should be proficient in the practice of basic clinical skills. However, a comparison of the performance in the Licensure Examination before and after the reform at both medical faculties in Munich (LMU und TU) showed a statistically significant decline of scores in the oral and practical part of the exam [22]. Changes in medical curricula in Germany and their impact on the increase in medical knowledge during clinical training are well studied [23], [24]; investigations of learned practical skills and achievement of competence based learning objectives are lacking. It is therefore unclear, how well undergraduate medical students receiving training more focused on clinical skills effectively master these skills in their final year.

Furthermore, the adequate self-assessment of performance and therefore one’s own limitations play a crucial role in the care for patients. Everyone involved in patient care should seek help in case of overload [25]. It is important for every (aspiring) physician to recognise limits of one’s own abilities and to prevent harm through erroneous action or even faulty omission. Danger lies in over-estimation (unconscious incompetence) as well as under-estimation (unconscious competence) of one’s abilities [26]. Multiple studies have shown that subjective self-assessment and objectively measured performance do not necessarily correlate [27], [28], [29]. Undergraduate medical students have a responsibility towards their patients as well as their teachers to estimate their skills adequately in order to improve on deficits and further develop strengths. This holds especially true as the physical examination acts as a cornerstone of diagnosis [2], [30]. Typically, final year medical students in Germany are first to see admitted patients. Therefore, a realistic self-assessment of examination proficiency is vital for the patients’ well-being and further course of hospital stay. We wanted to know how well undergraduate students in an advanced and critical part of their training could estimate their abilities to perform basic clinical skills.

## 2. Methods

Undergraduate medical students in their final year could participate in a formative oral and practical examination (“mündlich-praktische Prüfung im PJ”, abbreviated: mP3) using the OSCE format (objective structured clinical examination) from mid-2011 through 2014. The intent of this examination was to offer the participants the possibility to objectively assess practical clinical skills and obtain individual feedback as to identify strengths and weaknesses. The examination consisted of four OSCE stations. The stations covered various aspects of the physical examination: heart, lungs, abdomen, and vascular/lymphatic system as well as neurological, endocrinological, and orthopaedic examination. Amongst others, the stations covered basic clinical skills such as writing and reading of a 12-lead ECG, basic interpretation of an abdominal CT scan, identifying normal and abnormal findings on a chest X-ray, outlining the management of an emergency in the ER, enumerate important laboratory parameters to aid differential diagnosis in specified clinical settings). Two thirds of each station is devoted to the physical examination, the remaining third assesses other clinical skills. Each instalment of the OSCE consisted of stations compiled from a pool of 12 different OSCE stations. An expert panel designed and validated all stations. Participants performed the physical examination on probands instructed not to give any feedback during the examination. Marks for specific steps of the physical examination were awarded only if that step was performed correctly in its entirety. Each station lasted precisely 12 minutes. Afterwards, students obtained 3 minutes feedback from the examiner. All 19 examiners were faculty staff members with experience in examining OSCEs as well as professional experience as clinicians. Frequently held workshops for faculty by our Institute for Didactics and Education Research ensure a high standard of quality in the implantation of assessments such as the OSCE.

Participants voluntarily filled-out a questionnaire referring to personal and demographic details, the course of studies, prior training in healthcare (e.g. as paramedic or as nurse) as well as the assumed mark achieved in the examination (5 point scale as commonly used in Germany for school grading: exam mark 1 = “excellent” to 5 = “insufficient”). To allow for comparison of the self-assessment with the OSCE score (expressed as percentage of total achievable points), we converted the OSCE score into the same 5 point scale according to a conversion scheme common in Germany and used in the National License Examinations [8]. Students received a notification of their achieved score after the examination.

All statistical analyses were performed using SPSS (IBM Corp., Armonk, NY, U.S.A.). For the difference between two means, t-tests were used; in cases with multiple groups, an analysis of variance was performed. Effect sizes were assessed using Cohen’s d. p values α=0.05 were considered statistically significant.

The operational sequence, purpose, and intention of scientific interpretation of the data of this practical examination were announced to the local ethics committee, which deemed a formal ethical approval not necessary. The study was conducted according to principles of the World Medical Association’s Declaration of Helsinki and Declaration of Geneva. All undergraduate medical students in their final year could participate. Participation was voluntary and participants gave written consent to the scientific analysis of the examination and publication of results. Not consenting did not exclude students from the examination.

## 3. Results

### Study population

214 students participated in the study from mid-2011 until the end of 2014. Median age of participants was 26.3 (±4.5) years. Almost two thirds (64.0%; n=137) were female, thus corresponding to the gender distribution of all undergraduate medical students at the LMU Munich. There was no significant age difference between female and male participants (m=27.5±3.2 years; f=27.1±5.1 years; p=0.544). Most participants (n=156; 72.9%) had pursued their medical studies at the LMU Munich from the beginning; the other participants had joined the LMU Munich at later stages of their undergraduate studies.

#### Total performance

**Figure 1:**
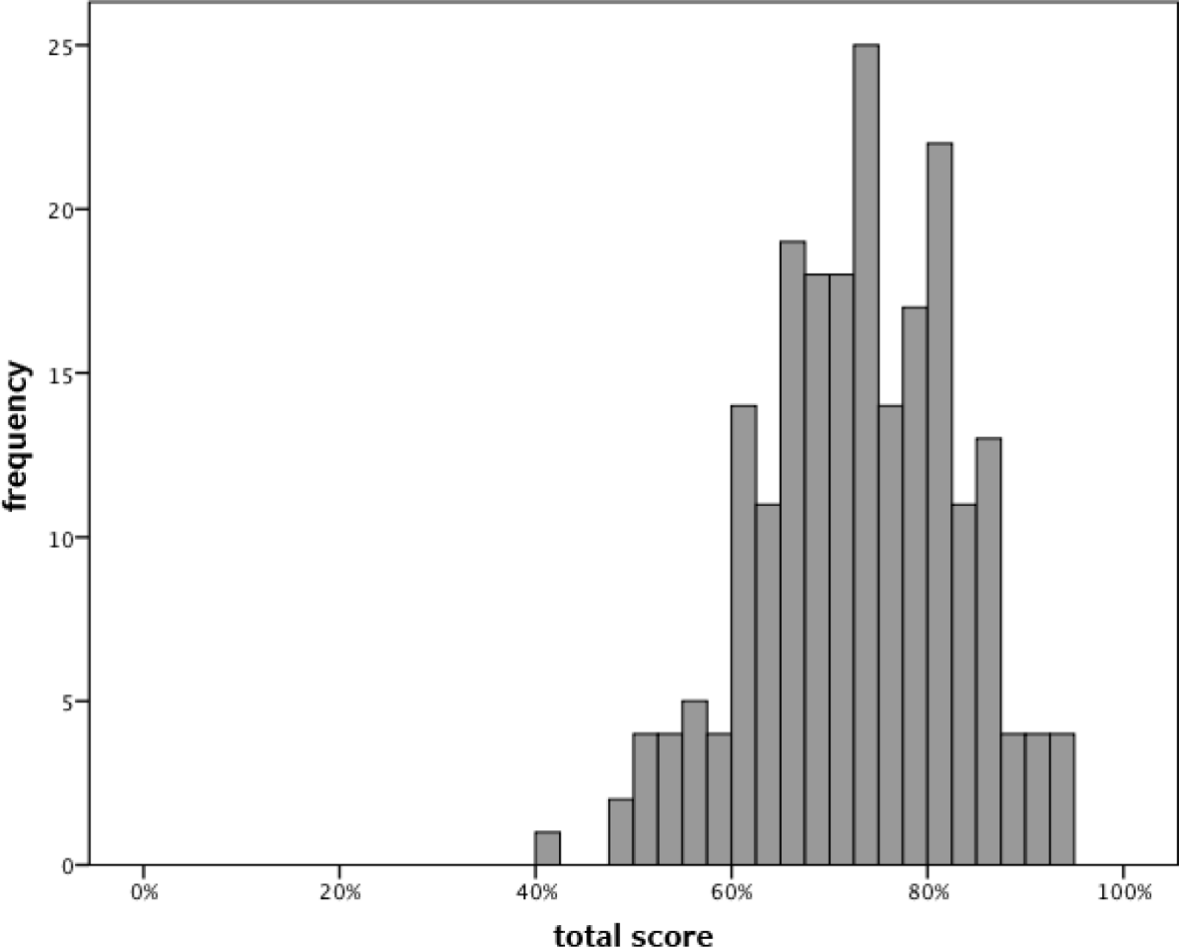
Histogram of total score (4-station OSCEs)

On average participants achieved 72.8%±10.1% of the maximum total score (see Figure 1 (Fig. 1)). After converting the performance score into a 5 point scale only 3.7% achieved an examination mark of “1” (“excellent”; score=90%; n=8), 23.4% a mark of “2” (“good”; score 80-90%; n=50), 34.6 a mark of “3” (“fair”; score 70-80%; n=74), and 29.0% a mark of “4” (“poor”; score 60-70%; n=62). Twenty students (9.3%) had an “insufficient” score (defined as<60%; mark of “5”).

#### Confounding factors

Female participants had a tendency towards slightly higher scores; however, this difference was not significant (73.7%±10.1% versus 71.1%±9.9%; p=0.069). There were no significant differences in scores between participants who had studied at the LMU Munich from the beginning vs. at later stages (p=0.349). A prior training in healthcare did not yield other scores than without prior training (p=0.363). Scores were homogenously distributed amongst participants from 2011 until 2014 (p=0.881).

The majority of participants stated not having prepared themselves specifically for the exam (63.1%). They achieved significantly lower scores in comparison to prepared students (71.8%±9.3% vs. 78.6%±10,0%; p<0,001; d=0,27). For an overview of these results, cf. Table 1 (Tab. 1).

#### Self-assessment

**Figure 2:**
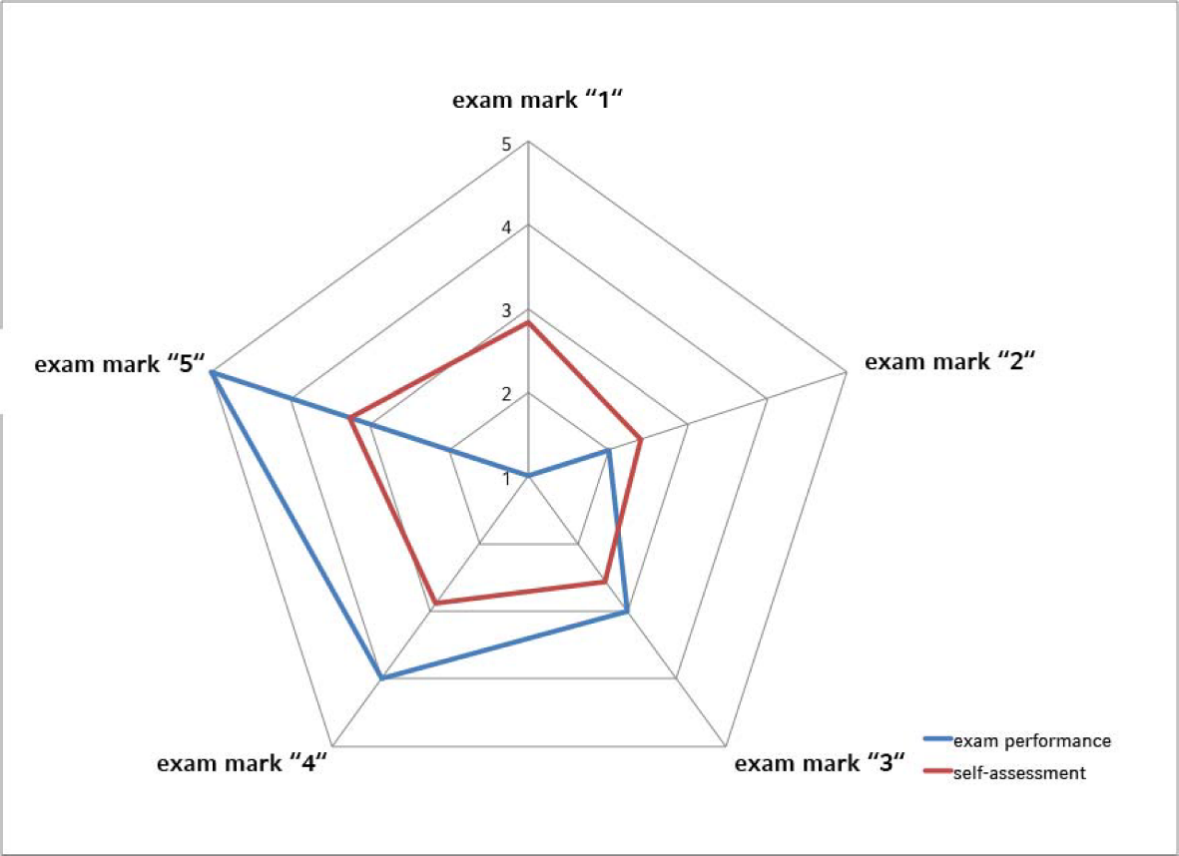
Radar chart of total examination performance (expressed as exam mark) and average self-assessment (same scale)

170 participants (79.4%) gave an estimate of their performance in the examination. Self-assessed performance and total examination score correlated positively and significantly (r=0.26; p<0.001). On average, students over-estimated their performance by half an examination mark. 60 participants (35.3%) correctly assessed their performance. 51 students (30.0%) over-estimated their performance by one, 32 participants (18.8%) by two marks. 21 students (12.4%) under-estimated their performance by one, 6 participants (3.5%) by two marks. Of the 20 participants with a total score below 60% (“insufficient”) 16 had self-assessed their performance of which 13 (81.3%) were over-estimating. Confer to Figure 2 (Fig. 2) for an overview of self-assessment in relation to total score.

## 4. Discussion

Practical clinical skills such as the physical examination remain an important instrument in the physician’s armamentarium. Our analysis of a formative, oral-practical examination in undergraduate medical students in their final year showed a lack of these skills despite the advanced course of studies and immanent licensure. Our participants had trouble performing a physical examination as well as basic clinical procedures such as writing and reading an ECG. A comparable analysis in American students during the USMLE Step 2 Clinical Skills Examination yielded similar results [31]. Recently Schmidmaier et al. used a progress test to show that knowledge of internal medicine continuously increases at the LMU Munich [24]. However, these results are not generalizable onto practical clinical skills [32].

In Germany acquiring new and improving on existing skills during the final year of undergraduate medical studies relies heavily on the supervision and patronage of the ward’s physicians where students spend their final year. In practice, supervision is lacking and the acquisition and improvement of skills depends largely on chance and the individual commitment of the students [33], [34], [35]. A rather new approach is to follow the development of clinical skills with a progress test longitudinally [36], [37], [38]; so far, published data are lacking.

Learning practical clinical skills requires complex interventions and a seamless interaction between all parties involved (medical faculty, teaching hospitals, and other hospitals/practices where students complete clinical traineeships). In reality, this is hardly controllable and students develop a large part of their “clinical practice” outside class [39]. Effectively this means that an important part of medical training is beyond the grasp of university structures and therefore escapes institutional quality standards. So far, teaching of practical clinical skills at faculty level focuses on the use of skills labs [40], [41] where peers mostly perform teaching (student tutors). Multiple studies have shown this concept to be effective [42], [43]. Structured formats improve practical clinical skills acquired in the skills lab lastingly [44]. Another mechanism is to perform intermittent formative examinations and make use of the “assessment drives learning” effect [45]. In respect to the data presented herein, it seems important to perform formative examinations assessing clinical skills as measures of quality assurance during the final year of undergraduate medical education [46]. Ideally, these examinations should be composed of assessments of diverse skills and compiled from an exhaustive catalogue [47]. In light of high costs of these examination formats [48], [49], faculties have to rethink how to find affordable solutions to improve the teaching of clinical skills, such as special tutorship programmes [50]. Important questions in this context are: Who profits from such formative examinations? How frequent should they be? At what point in time during the course of medical studies should the first examination take place? Is the OSCE the right format for such examinations? There are no answers to these questions derived from generalizable recommendations from the literature. To select specific students (and therefore to favour those) might seem ethically ill advised. However, additional interventions have proven helpful in those students at risk of attrition [51], [52]. From an economical stance, it could be justified to limit additional resources to those students at risk. A possible compromise (albeit increasing administrative overhead) could be to offer a certain minimum of such examinations to all students and to examine students at risk more frequently. How often and from which year of undergraduate education on is unclear as data is scarce [53]. We think that such examinations should begin early on to prevent giving feedback too late, i.e. when false manoeuvres have already become routine. Alternatives are other formats such as the Mini-CEX and others (CEC, DOPS) that can take place directly at the “workplace”. As such, they offer interesting possibilities to assess clinical skills intermittently with comparatively little à priori effort [54], [55], [56]. Conversely, more effort is required to instruct and train examiners correctly for these formats.

One third of all students correctly self-assessed their performance. Almost half of our participants over-estimated their performance; nearly one in five to a vast degree. This is more than previously described [29], [57]. The phenomenon is not new and neither limited to medical studies nor students per se [58], [59]. Our students received structured and qualitative feedback after the exanimation. Many students were surprised when they realized how off they were in their self-assessment. Consequences of over-estimation can be serious, in particular when it leads to diagnostic and/or therapeutic errors [60], [61], [62]. The ability to correctly self-evaluate is difficult to teach or train. It was postulated that video feedback would be sufficient to improve self-assessment [63]. A study by Hawkins and colleagues achieved improvement only retrospectively when a video of the students’ performance was juxtaposed a video demonstrating the correct manoeuvres [64]. It is therefore important to give the students a good idea of their performance through structured feedback, but also to show the correct execution of the skill assessed. Criticism must always be communicated in a meaningful way and it has to be noted that responsiveness to feedback is modulated by expectations and attitudes [65].

We presented data from a formative and voluntary examination, for which students had to register actively. This may have biased our results. One would expect eager and especially motivated students to participate in an optional examination leading to false-positive results. In so far we deem our data and the conclusions derived from them to be plausible. Our examination is well accepted and students use it to prepare themselves for the final part of the Licensure Examination, which also takes place as an oral and practical exam. A strength of our data is the size of the study population that allows for reliable statements even when effect size is small.

## 5. Conclusions

The performance of undergraduate medical students in their final year during a formative oral and practical clinical examination leaves ample room for improvement. Almost two thirds of our participants scored “fairly” or “poorly”; one in ten students fails. Practically half of our students over-estimated their own performance. An established, standardized, formative examination during the final year of undergraduate studies seems vital.

## Acknowledgements

We thank our examiners and colleagues for their support and all the efforts put into implementing the mP3 examination: Prof. Dr. Ralf Schmidmaier, PD Dr. Peter Reilich, PD Dr. Stefan Grote, PD Dr. Peter Thaller, PD Dr. Philipp Baumann, Dr. Kathrin Schrödl, Dr. Philip von der Borch, Dr. Bert Urban, Dr. Michael Maier, Dr. Mark op den Winkel, Dr. Costanza Chiapponi, Anja Fischer, Caroline Strenkert, Andreas Sturm, and Christina Berr.

## Competing interests

The authors declare that they have no competing interests.

## Figures and Tables

**Table 1:**
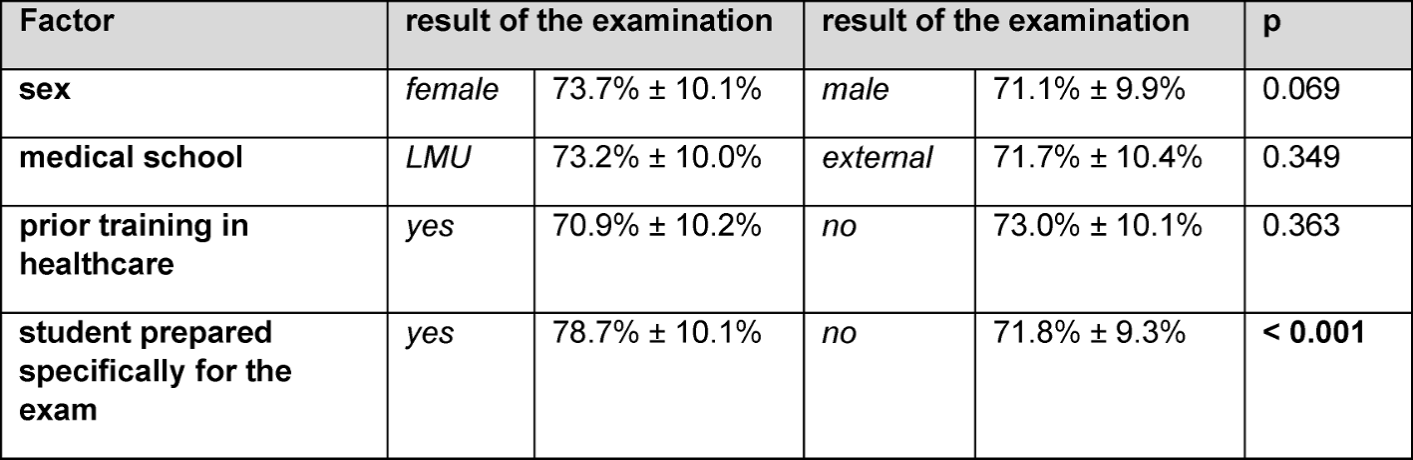
Possible confounding factors: comparison of total score between various sub-groups
